# The potential of probabilistic graphical models in linkage map construction

**DOI:** 10.1007/s00122-016-2824-x

**Published:** 2016-12-05

**Authors:** Huange Wang, Fred A. van Eeuwijk, Johannes Jansen

**Affiliations:** Biometris, Wageningen University and Research Centre, P.O. Box 16, 6700 AA Wageningen, The Netherlands

## Abstract

**Key message:**

**Probabilistic graphical models show great potential for robust and reliable construction of linkage maps. We show how to use probabilistic graphical models to construct high-quality linkage maps in the face of data perturbations caused by genotyping errors and reciprocal translocations.**

**Abstract:**

It has been shown that linkage map construction can be hampered by the presence of genotyping errors and chromosomal rearrangements such as inversions and translocations. Here, we report a novel method for linkage map construction using probabilistic graphical models. The method is proven, both theoretically and practically, to be effective in filtering out markers that contain genotyping errors. In particular, it carries out marker filtering and ordering simultaneously, and is therefore superior to the standard post hoc filtering using nearest-neighbour stress. Furthermore, we demonstrate empirically that the proposed method offers a promising solution to linkage map construction in the case of a reciprocal translocation.

**Electronic supplementary material:**

The online version of this article (doi:10.1007/s00122-016-2824-x) contains supplementary material, which is available to authorized users.

## Introduction

Genetic maps greatly facilitate a variety of genetic and genomic studies, including the genetic dissection of complex traits, comparative genomic analyses, and genome assembly (Bowers et al. 2012; Liu et al. 2014). Current approaches to map construction are mainly based on estimation of recombination frequency, and they aim to achieve three core objectives: (1) grouping, i.e. assigning markers to linkage groups; (2) ordering, i.e. finding the correct order of markers within each linkage group; and (3) spacing, i.e. estimating the map distances between pairs of adjacent markers (Cheema and Dicks 2009; Wu et al. 2008b).

Grouping is usually done by setting a threshold either directly on the pairwise recombination frequencies or on a statistic based on the pairwise recombination frequencies, e.g. the LOD scores (Van Os et al. 2005). Ordering can be viewed as an optimization problem. It typically involves two essential elements: (1) a scoring function that quantifies the quality of a given marker order, e.g. the likelihood (Cartwright et al. 2007; Jansen et al. 2001), the sum of adjacent recombination frequencies (SARF) (Falk 1989), the sum of adjacent LOD scores (SALOD) (Weeks and Lange 1987), the product of adjacent recombination fractions (PARF) (Wilson 1988) and weighted least squares (WLS) (Stam 1993); and (2) a search strategy that reduces the space of candidate marker orders, e.g. simulated annealing (Cartwright et al. 2007; Jansen et al. 2001), ant colony optimization (ACO) (Iwata and Ninomiya 2006), genetic algorithms (Gaspin and Schier 1998), evolutionary algorithms (Mester et al. 2003) or greedy and Lin–Kernighan heuristics (Van Os et al. 2005). The optimal marker order is the one that optimizes the scoring function. The map distance is measured in centiMorgan (cM), which is a unit that describes a recombination frequency of 1%. For complete data, spacing is straightforward once ordering is done (Wu et al. 2008b). For incomplete data, multi-point maximum likelihood estimates of recombination frequencies between adjacent markers can be obtained by the EM algorithm using the theory of hidden Markov models (Lander and Green 1987).

It has been recognized that genotyping errors tend to inflate map lengths and reduce the proportion of correctly ordered maps, particularly as marker density increases (Hackett and Broadfoot 2003; Shields et al. 1991). Markers exhibiting high nearest-neighbour stress (N.N.Stress, a quantity measuring the difference between estimated and observed recombination frequencies for the directly neighbouring loci with respect to a particular locus on the map) generally contain genotyping errors (Van Ooijen and Jansen 2013) and are, therefore, often removed from constructed genetic maps (Farré et al. 2011; Ting et al. 2013). Nonetheless, this post hoc filtering is inherently biased in terms of predictive validity, as it is applied to marker orders that are obtained under the assumption of no error.

Marker orders have been shown to be relatively robust against both missing data and genotyping errors for widely spaced markers (10 cM intervals) (Hackett and Broadfoot 2003). This essentially coincides with the proposition made in Vision et al. (2000). In their study, Vision et al. demonstrated that it is neither necessary nor desirable to genotype all markers in every individual of a large mapping population to get a high-density genetic map. Instead, genotyping a limited number of markers, which are evenly and sparsely distributed throughout the genome, is sufficient for constructing a high-confidence framework map. Afterwards, additional markers can be added to the framework map by certain fine-mapping strategies, so as to avoid the loss in map resolution.

Few methods have been proposed for linkage map construction in the case of reciprocal translocations. A reciprocal translocation refers to an even exchange of DNA fragments between two non-homologous chromosomes. Recombination between loci around the translocation breakpoints is severely suppressed. As a consequence, markers in these regions become ‘pseudo-linked’, i.e. markers that lie on different chromosomes involved in the translocation will be mapped onto a single linkage group (Farré et al. 2011).

Probabilistic graphical models (PGMs) combine graph theory and probability theory to give a multivariate statistical modelling framework. A PGM depicts a set of random variable as nodes or vertices in a graph, and encodes the conditional independence between variables through edges in the graph where a lack of an edge between two nodes indicates that the two variables are conditionally independent. Beyond existing successful applications of PGMs in the reconstruction of various biological networks (Airoldi 2007; Friedman 2004), we show here that they can also serve as a map construction method that does not suffer from wrong marker orders as a consequence of genotyping errors and reciprocal translocations. More specifically, we demonstrate both theoretically and empirically that linkage map construction using PGMs can achieve marker filtering and ordering at the same time effectively. Moreover, PGMs allow accurate positioning of the translocation breakpoint and correct ordering of markers on the distal parts of the two chromosomes.

## Materials and methods

### Partial correlation coefficient vs N.N.Stress in identifying markers having genotyping errors

The partial correlation coefficient provides a measure of conditional independence between variables, which forms the basis for construction of PGMs. Here, we demonstrate, theoretically, that the partial correlation coefficient can serve as an alternative to N.N.Stress to identify markers with genotyping errors. To begin with, a few basic concepts are briefly reviewed. The recombination frequency *θ* refers to the probability of observing a gamete with a recombinant haplotype in a single meiosis of a heterozygous parent. In this study, we mainly consider the recombination frequency between marker loci. For each marker, the two parental alleles are denoted by *a* and *b*, respectively. The genotyping error rate *ε* is the probability of observing allele *a* when *b* is the true allele, or vice versa. An observation on a set of markers is referred to as a phenotype. We investigate the probabilities associated with all possible phenotypes for an ordered triplet of markers *M*_1_–*M*_2_–*M*_3_ (Table 1). The genotypic frequencies are obtained under two assumptions:

**Table 1:** Genotypic frequencies for ordered triplet of markers *M*_1_–*M*_2_–*M*_3_. *θ*_*ij*_ (0 < *θ*_*ij*_ < 0.5) denote the recombination frequency between markers *M*_*i*_ and *M*_*j*_; $\varepsilon_{{M_{1} }}$, $\varepsilon_{{M_{2} }}$,and $\varepsilon_{{M_{3} }}$ denote locus-specific genotyping error rates, 0 < *ε* < 0.5

Marker type			Genotypic frequency ($\varepsilon_{{M_{1} }} = \,\varepsilon_{{M_{3} }} { = 0, }\,\varepsilon_{{M_{2} }} = \,\varepsilon$)
*M*_1_	*M*_2_	*M*_3_	
−1	−1	−1	0.5 × [(1 − *ε*)(1 − *θ*_12_)(1 − *θ*_23_) + *εθ*_12_ *θ*_23_]
−1	−1	1	0.5 × (1 − *ε*)(1 − *θ*_12_)*θ*_23_ + *εθ*_12_(1 − *θ*_23_)
−1	1	1	0.5 × *ε*(1 − *θ*_12_)*θ*_23_ + (1 − *ε*)*θ*_12_(1 − *θ*_23_)
−1	1	−1	0.5 × [*ε*(1 − *θ*_12_)(1 − *θ*_23_) + (1 − *ε*)*θ*_12_ *θ*_23_]
1	1	1	0.5 × [(1 − *ε*)(1 − *θ*_12_)(1 − *θ*_23_) + *εθ*_12_ *θ*_23_]
1	1	−1	0.5 × (1 − *ε*)(1 − *θ*_12_)*θ*_23_ + *εθ*_12_(1 − *θ*_23_)
1	−1	−1	0.5 × *ε*(1 − *θ*_12_)*θ*_23_ + (1 − *ε*)*θ*_*12*_(1 − *θ*_23_)
1	−1	1	0.5 × [*ε*(1 − *θ*_12_)(1 − *θ*_23_) + (1 − *ε*)*θ*_12_ *θ*_23_]

Numeric values −1 and 1 in the first three columns represent marker types *a* and *b*, respectively

1. Recombination events occurring in adjacent intervals are statistically independent;
2. the alleles *a* and *b* occur with equal probability (0.5).

For mathematical simplicity, we replace alleles *a* and *b* by the values −1 and 1, respectively. By doing so, the mean and variance of each marker, hereafter considered as a random variable and denoted by *M*_*k* (*k* = 1,2,3)_, become 0 and 1, respectively. This will greatly facilitate the derivations on (partial) correlation coefficients presented below.

#### Partial correlation coefficient

Under the settings mentioned above, the correlation coefficient between markers *M*_*i*_ and *M*_*j*_, *r*_*ij*_, is equal to the expectation value *E*[*M*_*i*_ × *M*_*j*_]. Let *θ*_ij_ (0 < *θ*_*ij*_ < 0.5) denote the recombination frequency between markers *M*_*i*_ and *M*_*j*_; $\varepsilon_{{M_{1} }}$, $\varepsilon_{{M_{2} }}$ and $\varepsilon_{{M_{3} }}$ denote locus-specific genotyping error rates. When $\varepsilon_{{M_{1} }} = \,\varepsilon_{{M_{3} }} { = 0}$ and $\varepsilon_{{M_{2} }} = \varepsilon$ (0 < *ε* < 0.5), we obtain

$$r_{12} = \left( {1{-}2\theta_{12} } \right)\left( {1{-}2\varepsilon } \right)$$

$$r_{23} = \left( {1{-}2\theta_{23} } \right)\left( {1{-}2\varepsilon } \right)$$

$$r_{13} = \left( {1{-}2\theta_{12} } \right)\left( {1{-}2\theta_{23} } \right).$$

It is obvious that (1 − 2*θ*_12_)(1 − 2*ε*) < 1 − 2*θ*_12_ and (1 − 2*θ*_23_)(1 − 2*ε*) < 1 − 2*θ*_23_. This shows that if *M*_2_ contains errors, *r*_12_ and *r*_23_ decrease when *ε* increases, whereas *r*_13_ remains unchanged.

The partial correlation coefficient $\rho_{{M_{i} M_{j} |M_{k} }}$ measures the correlation between markers *M*_*i*_ and *M*_*j*_ after removing the effect of marker *M*_*k*_. It can be computed as:

$$\rho_{{M_{i} M_{j} |M_{k} }} = \frac{{r_{ij} - r_{ik} \times r_{jk} }}{{\sqrt {1 - r_{ik}^{2} } \sqrt {1 - r_{jk}^{2} } }}.$$

It follows that

$$\rho_{{M_{1} M_{2} |M_{3} }} = \frac{{r_{12} - r_{13} \times r_{23} }}{{\sqrt {1 - r_{13}^{2} } \sqrt {1 - r_{23}^{2} } }},\quad \rho_{{M_{1} M_{2} |M_{3} }} = \frac{{r_{13} - r_{12} \times r_{23} }}{{\sqrt {1 - r_{12}^{2} } \sqrt {1 - r_{23}^{2} } }}.$$

We have derived that when $\varepsilon_{{M_{1} }} = \varepsilon_{{M_{3} }} = 0$ and $\varepsilon_{{M_{2} }}$ = *ε* (0 < *ε* < 0.5), $\rho_{{M_{1} M_{2} |M_{3} }}$ (and analogously, $\rho_{{M_{2} M_{3} |M_{1} }}$) is a monotonically decreasing function of *ε*, whereas $\rho_{{M_{1} M_{3} |M_{2} }}$ is a monotonically increasing function of *ε* (please refer to Supplementary Material for detailed derivation). This indicates that the association between a marker containing genotyping error and each of its flanking markers decreases with increasing error rate, whereas the association between the two flanking markers increases with increasing error rate.

In Table 2 we have summarized the values of *r*_12_, *r*_23_ and *r*_13_ with respect to eight different settings of $\varepsilon_{{M_{1} }}$, $\varepsilon_{{M_{2} }}$ and $\varepsilon_{{M_{3} }}$. Accordingly, we have derived the following relationships:

$$\rho_{{M_{1} M_{3} |M_{2} }} = \rho_{{\tilde{M}_{1} M_{3} |M_{2} }} = \rho_{{M_{1} \tilde{M}_{3} |M_{2} }} = \rho_{{\tilde{M}_{1} \tilde{M}_{3} |M_{2} }} = 0$$

$$0 < \rho_{{\tilde{M}_{1} \tilde{M}_{3} |\tilde{M}_{2} }} < \rho_{{\tilde{M}_{1} M_{3} |\tilde{M}_{2} }} = \rho_{{M_{1} \tilde{M}_{3} |\tilde{M}_{2} }} < \rho_{{M_{1} M_{3} |\tilde{M}_{2} }} < 1,\quad {\text{when}}\;\theta_{12} = \theta_{23}$$

$$0 < \rho_{{\tilde{M}_{1} \tilde{M}_{3} |\tilde{M}_{2} }} < \rho_{{\tilde{M}_{1} M_{3} |\tilde{M}_{2} }} < \rho_{{M_{1} \tilde{M}_{3} |\tilde{M}_{2} }} < \rho_{{M_{1} M_{3} |\tilde{M}_{2} }} < 1,\quad {\text{when}}\;\theta_{ 1 2} > \theta_{ 2 3}$$

$$0 < \rho_{{\tilde{M}_{1} \tilde{M}_{3} |\tilde{M}_{2} }} < \rho_{{M_{1} \tilde{M}_{3} |\tilde{M}_{2} }} < \rho_{{\tilde{M}_{1} M_{3} |\tilde{M}_{2} }} < \rho_{{M_{1} M_{3} |\tilde{M}_{2} }} < 1,\quad {\text{when}}\;\theta_{23} > \theta_{12} ,$$

where $M_{i}$ and $\tilde{M}_{i}$ denote a locus genotyped without and with error, respectively.

**Table 2:** Pairwise correlation coefficients for ordered triplet of markers *M*_*1*_–*M*_*2*_–*M*_*3*_

	*r*_12_	*r*_23_	*r*_13_
$\varepsilon_{{M_{1} }} = 0, \varepsilon_{{M_{2} }} { = 0, }\,\varepsilon_{{M_{3} }} { = 0}$	1 − 2*θ*_12_	1 − 2*θ*_23_	(1 − 2*θ*_12_)(1 − 2*θ*_23_)
$\varepsilon_{{M_{1} }} = \,\varepsilon , \, \varepsilon_{{M_{2} }} { = 0, }\,\varepsilon_{{M_{3} }} { = 0}$	(1 − 2*θ*_12_)(1 − 2*ε*)	1 − 2*θ*_23_	(1 − 2*θ*_12_)(1 − 2*θ*_23_)(1 − 2*ε*)
$\varepsilon_{{M_{1} }} { = 0, }\,\varepsilon_{{M_{2} }} { = 0, }\,\varepsilon_{{M_{3} }} = \,\varepsilon$	1 − 2*θ*_12_	(1 − 2*θ*_23_)(1 − 2*ε*)	(1 − 2*θ*_12_)(1 − 2*θ*_23_)(1 − 2*ε*)
$\varepsilon_{{M_{1} }} = \,\varepsilon , \, \varepsilon_{{M_{2} }} { = 0, }\,\varepsilon_{{M_{3} }} = \,\varepsilon$	(1 − 2*θ*_12_)(1 − 2*ε*)	(1 − 2*θ*_23_)(1 − 2*ε*)	(1 − 2*θ*_12_)(1 − 2*θ*_23_)(1 − 2*ε*)^2^
$\varepsilon_{{M_{1} }} { = 0, }\,\varepsilon_{{M_{2} }} = \,\varepsilon , { }\,\varepsilon_{{M_{3} }} { = 0}$	(1 − 2*θ*_12_)(1 − 2*ε*)	(1 − 2*θ*_23_)(1 − 2*ε*)	(1 − 2*θ*_12_)(1 − 2*θ*_23_)
$\varepsilon_{{M_{1} }} = \,\varepsilon , \, \varepsilon_{{M_{2} }} = \,\varepsilon , { }\,\varepsilon_{{M_{3} }} { = 0}$	(1 − 2*θ*_12_)(1 − 2*ε*)^2^	(1 − 2*θ*_23_)(1 − 2*ε*)	(1 − 2*θ*_12_)(1 − 2*θ*_23_)(1 − 2*ε*)
$\varepsilon_{{M_{1} }} = 0, \varepsilon_{{M_{2} }} = \varepsilon , \varepsilon_{{M_{3} }} = \varepsilon$	(1 − 2*θ*_12_)(1 − 2*ε*)	(1 − 2*θ*_23_)(1 − 2*ε*)^2^	(1 − 2*θ*_12_)(1 − 2*θ*_23_)(1 − 2*ε*)
$\varepsilon_{{M_{1} }} = \,\varepsilon , \, \varepsilon_{{M_{2} }} = \,\varepsilon , { }\varepsilon_{{M_{3} }} = \,\varepsilon$	(1 − 2*θ*_12_)(1 − 2*ε*)^2^	(1 − 2*θ*_23_)(1 − 2*ε*)^2^	(1 − 2*θ*_12_)(1 − 2*θ*_23_)(1 − 2*ε*)^2^

Denotations of *θ*_12_, *θ*_23_, $\varepsilon_{{M_{1} }}$, $\varepsilon_{{M_{2} }}$, $\varepsilon_{{M_{3} }}$ and *ε* are identical to those in Table 1

#### N.N.Stress

Genotyping errors that occur at a marker will increase the observed recombination frequencies between that marker and its flanking markers (Goring and Terwilliger 2000). When $\varepsilon_{{M_{1} }} = \varepsilon_{{M_{3} }} = 0$ and $\varepsilon_{{M_{2} }} = \varepsilon$ (0 < *ε* < 0.5),

$$\rho_{12} = \theta_{12} + \varepsilon \left( {1{-}2\theta_{12} } \right)$$

$$\rho_{23} = \theta_{23} + \varepsilon \left( {1{-}2\theta_{23} } \right)$$

$$\rho_{13} = \theta_{12} + \theta_{23} {-}2\theta_{12} \theta_{23} ,$$

where $\rho_{ij}$ denote the observed recombination frequency between two markers *M*_*i*_ and *M*_*j*_. Let *d*_*ij*_ denote the distance (in Morgans) between two markers *M*_*i*_ and *M*_*j*_. Applying Haldane’s mapping function, *d*_*ij*_ = –0.5ln(1 − 2 $\rho_{ij}$), gives

$$d_{12} = {-}0.5\ln [(1{-}2\theta_{12} )(1{-}2\varepsilon )]$$

$$d_{23} = {-}0.5\ln [(1{-}2\theta_{23} )(1{-}2\varepsilon )]$$

$$d_{13} = {-}0.5\ln [(1{-}2\theta_{12} )(1{-}2\theta_{23} )].$$

The N.N.Stress of marker *M*_*2*_ given *M*_*1*_ and *M*_*3*_ is computed as:

$$d_{12} + d_{23} {-}d_{13} = {-}\ln \left( {1{-}2\varepsilon } \right).$$

Given that 0 < *ε* < 0.5, −ln(1 − 2*ε*) is a monotonically increasing function of *ε*. This indicates that markers genotyped with high error rate exhibit large N.N.Stress.

Analogously, we have investigated and listed the values of $\rho_{ 1 2}$, $\rho_{ 2 3}$ and $\rho_{ 1 3}$ with respect to eight different settings of $\varepsilon_{M1}$, $\varepsilon_{{M_{2} }}$ and $\varepsilon_{{M_{3} }}$ in Table 3. Further, we have derived the relationships below:

$${\text{N}} . {\text{N}} . {\text{ Stress}}_{{M_{2} |M_{1} ,M_{3} }} = {\text{N}} . {\text{N}} . {\text{ Stress}}_{{M_{2} |\tilde{M}_{1} ,M_{3} }} = {\text{N}} . {\text{N}} . {\text{ Stress}}_{{M_{2} |M_{1} ,\tilde{M}_{3} }} = {\text{N}} . {\text{N}} . {\text{ Stress}}_{{M_{2} |\tilde{M}_{1} ,\tilde{M}_{3} }} = 0,$$

$$0 < {\text{N}} . {\text{N}} . {\text{ Stress}}_{{\tilde{M}_{2} |\tilde{M}_{1} ,\tilde{M}_{3} }} = {\text{N}} . {\text{N}} . {\text{ Stress}}_{{\tilde{M}_{2} |\tilde{M}_{1} ,M_{3} }} = {\text{N}} . {\text{N}} . {\text{ Stress}}_{{\tilde{M}_{2} |M_{1} ,\tilde{M}_{3} }} = {\text{N}} . {\text{N}} . {\text{ Stress}}_{{\tilde{M}_{2} |M_{1} ,M_{3} }} = {-}\ln \left( {1{-}2\varepsilon } \right),$$

where ${\text{N}} . {\text{N}} . {\text{Stress}}_{{M_{j} |M_{i} ,M_{k} }}$ denote the N.N.Stress of *M*_j_ given its flanking markers *M*_*i*_ and *M*_*k*_.

**Table 3:** The observed pairwise recombination frequencies for ordered triplet of markers *M*_1_–*M*_2_–*M*_3_

	$\rho_{ 1 2}$	$\rho_{ 2 3}$	$\rho_{ 1 3}$
$\varepsilon_{{M_{1} }} = 0, \, \varepsilon_{{M_{2} }} = 0, \varepsilon_{{M_{3} }} { = 0}$	*θ*_12_	*θ*_23_	*θ*_12_ + *θ*_23_ − 2*θ*_12_ *θ*_23_
$\varepsilon_{{M_{1} }} = \varepsilon , \, \varepsilon_{{M_{2} }} = 0, \varepsilon_{{M_{3} }} { = 0}$	*θ*_12_ + *ε*(1 − 2*θ*_12_)	*θ*_23_	*θ*_12_ + *θ*_23_ − 2*θ*_12_ *θ*_23_ + *ε*(1 − 2*θ*_12_)(1 − 2*θ*_23_)
$\varepsilon_{{M_{1} }} = 0, \, \varepsilon_{{M_{2} }} = 0, \varepsilon_{{M_{3} }} = \varepsilon$	*θ*_12_	*θ*_23_ + *ε*(1 − 2*θ*_23_)	*θ*_12_ + *θ*_23_ − 2*θ*_12_ *θ*_23_ + *ε*(1 − 2*θ*_12_)(1 − 2*θ*_23_)
$\varepsilon_{{M_{1} }} = \varepsilon , \, \varepsilon_{{M_{2} }} = 0, \varepsilon_{{M_{3} }} = \varepsilon$	*θ*_12_ + *ε*(1 − 2*θ*_12_)	*θ*_23_ + *ε*(1 − 2*θ*_23_)	*θ*_12_+ *θ*_23_ − 2*θ*_12_ *θ*_23_ + 2*ε*(1 − *ε*)(1 − 2*θ*_12_)(1 − 2*θ*_23_)
$\varepsilon_{{M_{1} }} = 0, \, \varepsilon_{{M_{2} }} = \varepsilon , \varepsilon_{{M_{3} }} { = 0}$	*θ*_12_ + *ε*(1 − 2*θ*_12_)	*θ*_23_ + *ε*(1 − 2*θ*_23_)	*θ*_12_ + *θ*_23_ − 2*θ*_12_ *θ*_23_
$\varepsilon_{{M_{1} }} = \varepsilon , \, \varepsilon_{{M_{2} }} = \varepsilon , \varepsilon_{{M_{3} }} { = 0}$	*θ*_12_ + 2*ε*(1 − *ε*)(1 − 2*θ*_12_)	*θ*_23_ + *ε*(1 − 2*θ*_23_)	*θ*_12_ + *θ*_23_ − 2*θ*_12_ *θ*_23_ + *ε*(1 − 2*θ*_12_)(1 − 2*θ*_23_)
$\varepsilon_{{M_{1} }} = 0 , \, \varepsilon_{{M_{2} }} = \varepsilon , \varepsilon_{{M_{3} }} = \varepsilon$	*θ*_12_ + *ε*(1 − 2*θ*_12_)	*θ*_23_ + 2*ε*(1 − *ε*)(1 − 2*θ*_23_)	*θ*_12_ + *θ*_23_ − 2*θ*_12_ *θ*_23_ + *ε*(1 − 2*θ*_12_)(1 − 2*θ*_23_)
$\varepsilon_{{M_{1} }} = \varepsilon , \, \varepsilon_{{M_{2} }} = \varepsilon , \varepsilon_{{M_{3} }} = \varepsilon$	*θ*_12_ + 2*ε*(1 − *ε*)(1 − 2*θ*_12_)	*θ*_23_ + 2*ε*(1 − *ε*)(1 − 2*θ*_23_)	*θ*_12_ + *θ*_23_ − 2*θ*_12_ *θ*_23_ + 2*ε*(1 − *ε*)(1 − 2*θ*_12_)(1 − 2*θ*_23_)

Denotations of *θ*_*12*_, *θ*_*23*_, $\varepsilon_{{M_{1} }}$, $\varepsilon_{{M_{2} }}$, $\varepsilon_{{M_{3} }}$ and *ε* are identical to those in Table 1

In view of the similarity between relationships revealed by partial correlation and N.N.Stress, we are able to draw the following conclusions:

1. When the marker data contain no genotyping errors, the partial correlations between physically non-adjacent markers are all equal to 0, whereas the absolute partial correlations between physically adjacent markers are close to 1. It implies that, ideally, marker ordering can be carried out through diagonalization of the partial correlations matrix.
2. In addition to its application to marker ordering, partial correlation coefficient can also serve as an alternative to N.N.Stress, i.e. it can be used to identify markers involving genotyping errors. More specifically, if *ρ*_*M1M3*|*M2*_ is larger than a certain threshold, for the conditioning marker *M*_2_ one of the two situations holds:
  (i) *M*_2_ is not genetically located between *M*_1_ and *M*_3_;
  (ii) *M*_2_ is indeed between *M*_1_ and *M*_3_, but contains genotyping error (alternatively, the error rate of *M*_2_ is much greater than the error rates of *M*_1_ and *M*_3_).
    Notably, a large $\rho_{{M_{1} M_{3} |M_{2} }}$ always comes with small *r*_12_ and *r*_23_, which indicates, in the context of PGMs, that *M*_1_ and *M*_3_ are, highly likely, directly connected to each other, whereas *M*_2_ is, quite possibly, disconnected from *M*_1_ and *M*_3_. This naturally provides a simultaneous graphical representation of two situations:
  (i) The non-intermediate marker *M*_2_ is excluded from the connection between *M*_1_ and *M*_3_;
  (ii) The intermediate marker *M*_2_ that involves big genotyping error is excluded from the connection between *M*_1_ and *M*_3_.
3. If not only *M*_2_ but also *M*_1_ or/and *M*_3_ have genotyping errors (alternatively, the error rates of *M*_2_, *M*_1_ or/and *M*_3_ are comparable), the increment of *ρ*_*M*1*M*3|*M*2_ decreases while ${\text{N}} . {\text{N}} . {\text{Stress}}_{{M_{2}|M_{1},M_{3} }}$ does not change. This suggests in the application of partial correlation for identifying markers with genotyping errors, smaller cutoff values are preferable so that minor increases caused by genotyping errors of at least two markers in a triplet can still be captured.
4. When *M*_2_ has no genotyping error, there is no increase of $\rho_{{M_{1} M_{3} |M_{2} }}$, despite of genotyping errors occurring on either or both of *M*_1_ and *M*_3_. This shows partial correlation is limited to filtering out markers that simultaneously satisfy three requirements:
  (i) They are taken as conditioning variables;
  (ii) they are intermediates in triplets of markers;
  (iii) they have high error rates.

However, this limitation can be overcome by iterative implementation of partial correlation estimation on sequential triplets of markers. Specifically, assume that *M*_1_–*M*_2_–*M*_3_–*M*_4_ is the true order of four markers, of which *M*_3_ has a high error rate. Then, the problematic marker *M*_3_ can be filtered out by investigating $\rho_{{M_{2} M_{4} |M_{3} }}$ instead of $\rho_{{M_{1} M_{3} |M_{2} }}$.

### The PC-stable algorithm

In the construction of PGMs, the conditional independence relationships among a set of variables are typically represented in the form of an undirected graph. The PC algorithm (Spirtes et al. 2000) was originally designed to learn a Markov equivalence class of directed acyclic graphs that can be uniquely described as a completed partially directed acyclic graph (CPDAG) (Hauser and Buhlmann 2012). Its learning process consists of two phases: first, construct an undirected graph by means of a series of well-structured conditional independence tests; and second, assign directions to certain edges according to the determined v-structures and the acyclic constraint, so that the undirected graph is transformed into a CPDAG.

It should be noted that only the first phase of the PC algorithm is applicable to linkage map construction, since in such a context the directionality of edges between markers is meaningless. However, it has been pointed out that the first phase of the PC algorithm returns order-dependent skeletons (Colombo and Maathuis 2014). That is, the resulting undirected graph is subject to the order of variables present in the input data. For this reason, a modified version of the PC algorithm, which is referred to as the PC-stable algorithm, has been presented to overcome the order-dependent issue (Colombo and Maathuis 2014). The PC-stable algorithm is implemented in the R package *pcalg*.

### Frequentist diagonal ordering

In the application of the PC-stable algorithm to linkage mapping, the resulting undirected graphs usually capture the connectivity of markers to a large extent. Nonetheless, the linearity of markers could be a bit ambiguous at certain detailed parts. To eliminate such minor ambiguities, here we have proposed a frequentist diagonal ordering algorithm, which serves as a complement to the PC-stable algorithm for fine-ordering of markers. The logic behind this algorithm is rather straightforward: first, represent the undirected graph achieved by the PC-stable algorithm in the form of an adjacency matrix, which is typically a (0,1)-matrix with entries “1” indicating that the corresponding two (row & column) variables are directly connected in the graph; second, restructure the adjacency matrix so that as many “1” entries as possible are located on the first super diagonal of the new adjacency matrix; and third, convert the new adjacency matrix into input of a network visualization tool (e.g. Cytoscape), and let the relationships between markers be presented graphically. Essentially, this algorithm is to extract a marker string, as long as possible, from the constructed PGM. The related Matlab source code is available at: https://github.com/Huange/Frequentist-diagonal-ordering.

### Simulated data

A doubled-haploid population was simulated using the R package hypred. Two homozygous parental lines with genotypes *aa* and *bb* at each of 200 loci, which were evenly distributed along a single chromosome of 300 cM, were simulated initially. The two parental lines were then crossed to give an F1 population with heterozygous genotype *ab* at each locus. Subsequently, 300 doubled-haploid individuals were simulated from the gametes produced by the F1 generation. No interference was simulated, and so Haldane’s mapping function was applicable to the marker data. The markers were numerically labelled from 1 to 200 according to their relative positions along the chromosome. Among them, six markers, 34, 51, 63, 128, 155 and 184, were set to have genotyping errors at rates of 1, 3, 5, 1, 3 and 5%, respectively.

### Cucumber data

This set of marker data was obtained from an RIL population derived from an inter-subspecific cross between the North American processing market type cucumber cultivar Gy14 (*C. sativus* var. s*ativus*) and the wild accession PI 183967 (*C. sativus* var. hardwickii) originating from India. The RIL population consisted of 77 *F*_6_–*F*_8_ individuals, each of which was genotyped with 995 SSR markers. For more details, see Ren et al. (2009). To deal with missing values in the marker scores, we used a hidden Markov model approach (Jiang and Zeng 1997) implemented in Genstat to estimate the marker genotypes. It appeared interesting to investigate these data because our pre-processing results showed that genotyping errors were widely present across the whole dataset; besides, redundant markers existed in the sense that some markers were located on more or less the same locus.

### Barley data

This set of marker data is obtained from DH_1_ population developed from a cross between the barley varieties ‘Albacete’ and ‘Barberousse’. ‘Albacete’ is known for containing a reciprocal translocation between chromosomes 1H and 3H. The dataset consisted of 231 lines and 30 markers, of which 13 markers were located on chromosome 1H and 17 markers on chromosome 3H. For more details, see Farré et al. (2011).

## Results

### Simulated data

**Fig. 1:**
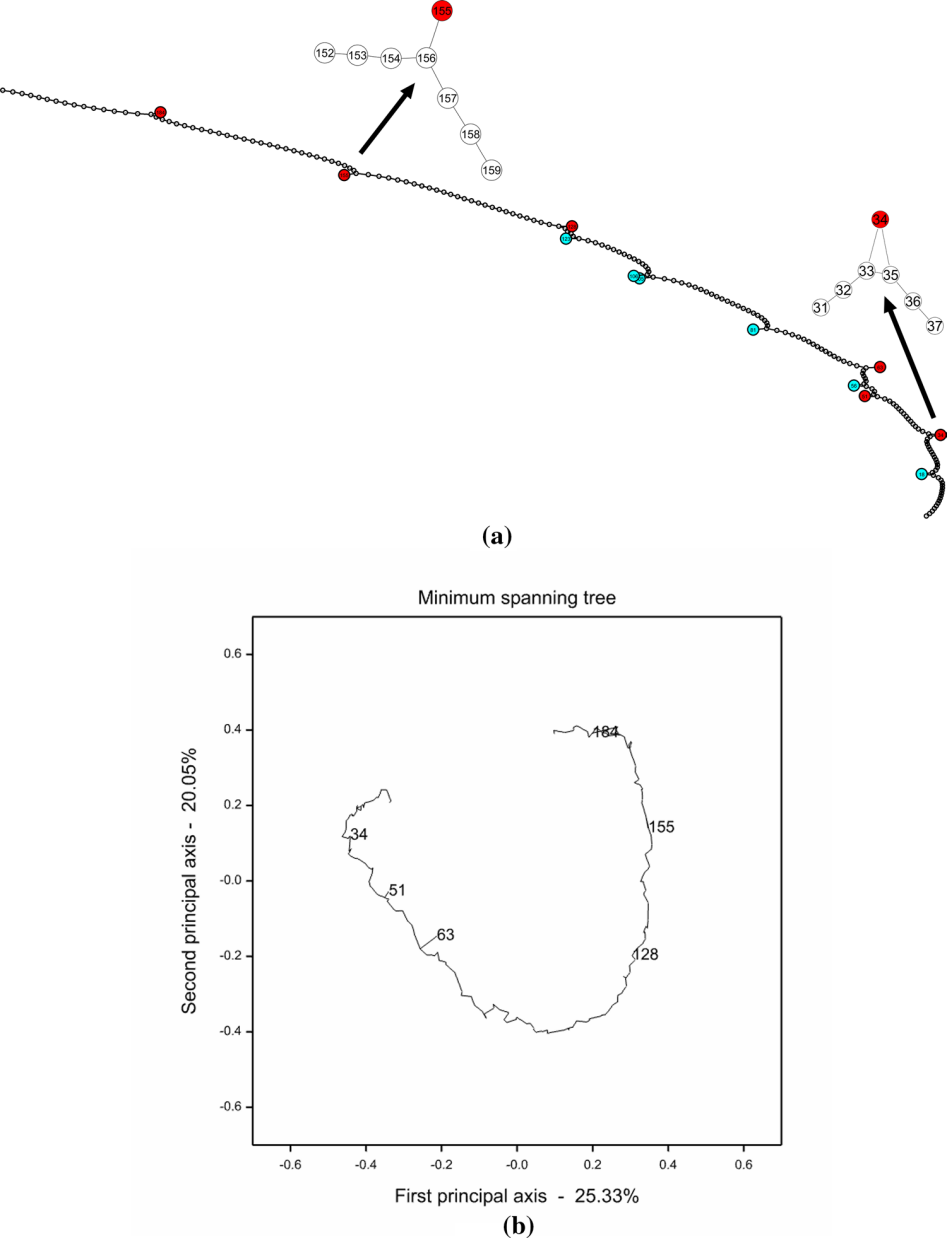
**a** A PGM constructed with the PC-stable algorithm for the simulated data. The six markers designed with genotyping errors are pulled aside from the linear string and coloured in *red*, and another six markers pulled aside from the linear string are coloured in *cyan*. Enlargements of two detailed parts of the PGM are given above the linear string, though the whole graph itself can be enlarged dramatically to show all details clearly. **b** An MST constructed with Genstat for the simulated data. The diagram was projected on the first two principal axes obtained by a principal coordinate analysis. Only the six markers designed with genotyping errors are marked out and coloured in *red* (colour figure online)

By applying the PC-stable algorithm to the simulated marker data, we obtained a linkage map as shown in Fig. 1a. In the map, all the six markers having genotyping errors (i.e. nodes coloured in red) were successfully identified, as they were pulled aside from the linear string formed by the vast majority of all other markers. Meanwhile, a couple of markers without genotyping errors (i.e. nodes coloured in cyan) were also pulled aside from the linear string. This should be attributed to the inherently weak connectivity between those markers and their flanking markers.

For comparison, we also reconstructed a linkage map from the simulated data with JoinMap 4.1. Table S1 gives the map position and the N.N.Stress of each marker in cM. It shows that the 200 markers were perfectly sequentially ordered, though a few markers possessed high N.N.Stress and, thus, should be removed from the reconstructed map. Table 4 lists the top six markers with the highest N.N.Stress, which are, as expected, exactly those markers designed with genotyping errors. Furthermore, a minimum spanning tree (MST) was constructed with Genstat from the same dataset (Fig. 1b), since MST has been claimed as another promising tool for efficient and accurate reconstruction of linkage maps (Wu et al. 2008b). Similarly, the 200 markers were substantially linearly arranged in the MST, except that only three markers 51, 63 and 184 were clearly shown in branches, indicating that they should be excluded from the reconstructed linkage map.

**Table 4:** The top six markers with the highest N.N.Stress obtained by JoinMap 4.1 from the simulated marker data

	Locus	Position	N.N.Stress (cM)
1	Marker63	96.145	11.01
2	Marker184	296.883	10.939
3	Marker155	243.352	6.318
4	Marker51	70.273	5.629
5	Marker128	195.223	2.046
6	Marker34	43.34	2.041

### Cucumber data

#### Data pre-processing

In a single seed descent (SSD) procedure, the percentage of heterozygotes is halved in each generation. In the cucumber data, the proportion of heterozygotes was according to the expectation for most individuals, but high for about 10% of the individuals (Fig.S1). Considering that the intention of SSD is to make all heterozygotes disappear eventually, we made all heterozygous scores missing and treated the entire population as an RIL_∞_ population, i.e. an RIL population obtained after infinitely many generations of SSD. This might lead to some individuals with a high proportion of missing data. Afterwards, we first excluded markers with more than four (>5.2%) missing data. This concerned 132 markers, leaving 863 markers for further analysis. We then excluded individuals with >10% missing data. These concerned only two individuals, leaving 75 individuals for further analysis. It should be noted that the number of individuals is small for accurate map construction.

#### Forming linkage groups

With a threshold of 0.2 for the recombination frequency, two linkage groups were formed, consisting of 719 and 144 markers, respectively. With a threshold of 0.15, the linkage group consisting of 144 markers remained intact, while the linkage group consisting of 719 markers was split into five subgroups, consisting of 340, 108, 107, 95 and 69 markers, respectively. With a threshold of 0.10, the linkage group consisting of 340 markers was further split into three groups of 177, 162 and 1 markers, respectively. With a threshold of 0.10, also the linkage group consisting of 69 markers was split into three groups of 38, 30 and 1 markers, respectively. Given the estimated six linkage groups obtained with a threshold of 0.15, we used the ML algorithm of JoinMap (Stam 1993) five times to check the stability of the resulting genetic maps. The results indicated that only the linkage group consisting of 340 markers should be split into two groups (Fig. S2): the first 177 markers at the upper part of the map (0–350 cM), and the remaining 163 markers at the lower part of the map (370–750 cM). The reason is that although there was a small gap between the two groups, there was no exchange of markers between the two groups in repeated runs of the ML algorithm. In summary, the 863 markers could be divided into seven linkage groups consisting of 177, 163, 144, 108, 107, 95 and 69 markers, respectively. Notably, this grouping was consistent with the one shown by the data providers, who assigned indicators Chr.6, Chr.3, Chr.5, Chr.2, Chr.1, Chr.4 and Chr.7 to the seven linkage groups, respectively. Table 5 offers, for each linkage group, a summary of the total number of markers, the number of unique markers, average map length across five mapping runs, and the highest value of N.N.Stress. The lengths of the preliminary maps constructed for each linkage group were fairly consistent over five mapping runs. Nonetheless, they were always large and especially so for Chr.3 and Chr.6. Also, the highest N.N.Stress is generally quite high. Both phenomena are indicators of genotyping errors in the marker data. Genotyping errors inflate pairwise recombination frequencies between markers (Goring and Terwilliger 2000), and subsequently inflate map lengths and harm the accuracy of marker ordering (Hackett and Broadfoot 2003; Shields et al. 1991).

**Table 5:** A summary of the total number of markers, the number of unique markers, the average map length across five mapping runs, and the highest values of N.N.Stress for each of the seven linkage groups constructed from the cucumber data (before missing data imputation)

Linkage group	Number of markers	Number of unique markers	Average map length in 5 runs (cM)	Highest N.N.Stress (cM)
Chr.1	107	103	195.1	8.0
Chr.2	108	103	269.8	11.7
Chr.3	163	151	343.9	22.3
Chr.4	95	67	115.4	11.8
Chr.5	144	104	155.0	9.1
Chr.6	177	157	333.1	12.0
Chr.7	69	66	176.7	9.5
Total	863	751		

In this study, we will focus on the map construction for Chr.5, which is an example involving issues of genotyping errors in combination with locally high marker density. The original 144 markers of Chr.5 contained 104 markers, which were unique when accounting for the pattern of missing data alongside with the observed marker phenotypes. After missing data imputation 64 unique markers remained. Hereafter, we will use the imputed data of the 64 markers (but the marker numbers refer to the set of 104 markers) to illustrate our method.

#### Identifying representative markers having genotyping errors for Chr.5

Initially, we focused on a subset of 20 markers that were representative of Chr.5. The 20 markers were obtained as cluster centres of a K-medoids clustering as implemented in the QMKSELECT procedure of Genstat. According to the expectation, the cluster centres should either be:

(i) High-quality markers (i.e. markers virtually without errors), in which case markers assigned to be a cluster are similar to the cluster centre, with a few more errors;
(ii) low-quality markers (i.e. markers with many errors), in which case the cluster is equivalent to its centre. Indeed, we observed that some of the 20 markers only represented themselves, that is, clusters of size 1.

**Fig. 2:**
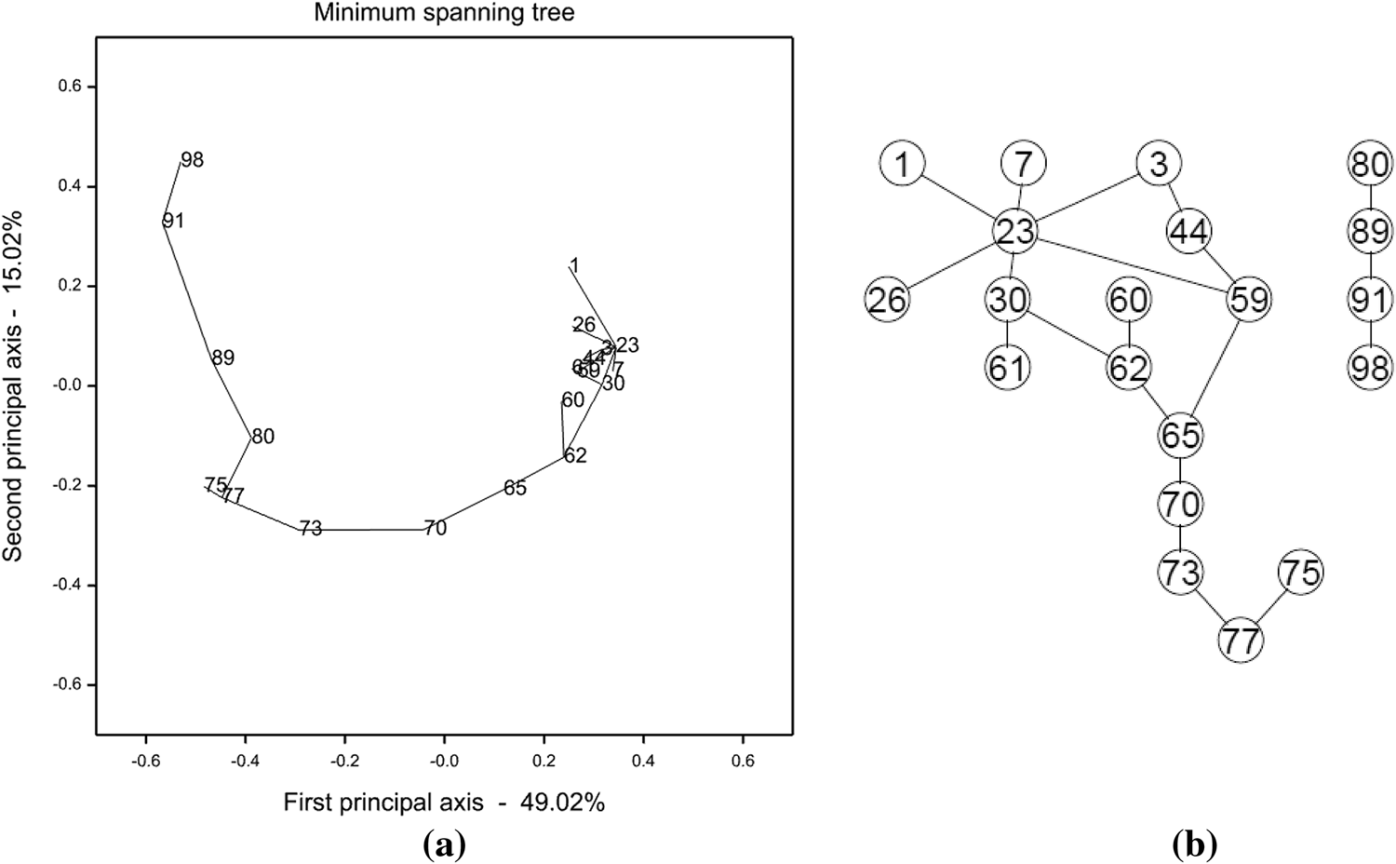
**a** An MST constructed with Genstat for 20 representative markers of Chr.5. The diagram was projected on the first two principal axes obtained by a principal coordinate analysis. **b** A PGM constructed with the PC-stable algorithm for the same set of 20 markers. The significance level for conditional independence tests was set at 0.05

For the 20 markers, we constructed an MST with Genstat and a PGM with the PC-stable algorithm, respectively (Fig. 2; a linearized version of the MST is shown in Fig. S3). Most links present in the two graphs were consistent, except that markers 44 and 59, 59 and 65 were connected while markers 77 and 80 were disconnected in the PGM. We also constructed a series of linkage maps with JoinMap 4.1 by sequentially deleting the markers with the highest, positive N.N.Stress (Fig. S4). The deleted markers shown at the top of Fig.S4 were almost identical to the problematic markers revealed in Fig. 2, i.e. markers deviating from the linear tree. Notably, N.N.Stress analysis indicated that marker 77 had large genotyping error and thus should be excluded from an accurate linkage map. In this regard, the obtained PGM is considered a bit more precise than the MST, since in the former a string of markers was disconnected from marker 77, whereas the latter did not uncover the error issue underlying marker 77.

#### Constructing framework map for Chr.5

**Fig. 3:**
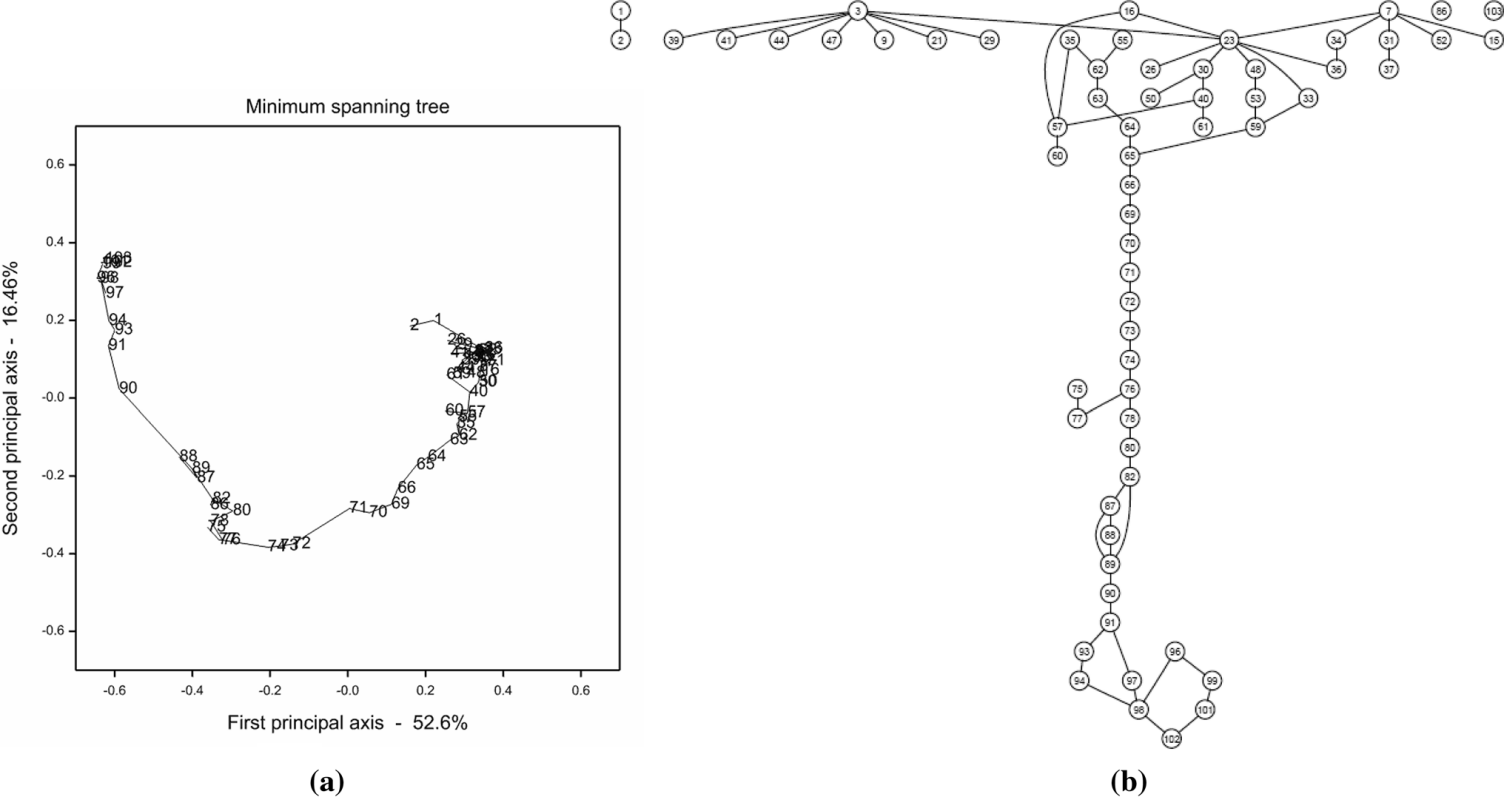
**a** An MST constructed with Genstat for 64 unique markers of Chr.5. The diagram was projected on the first two principal axes obtained by a principal coordinate analysis. **b** A PGM constructed with the PC-stable algorithm for the same set of 64 markers. The significance level for conditional independence tests was set at 0.05

**Fig. 4:**
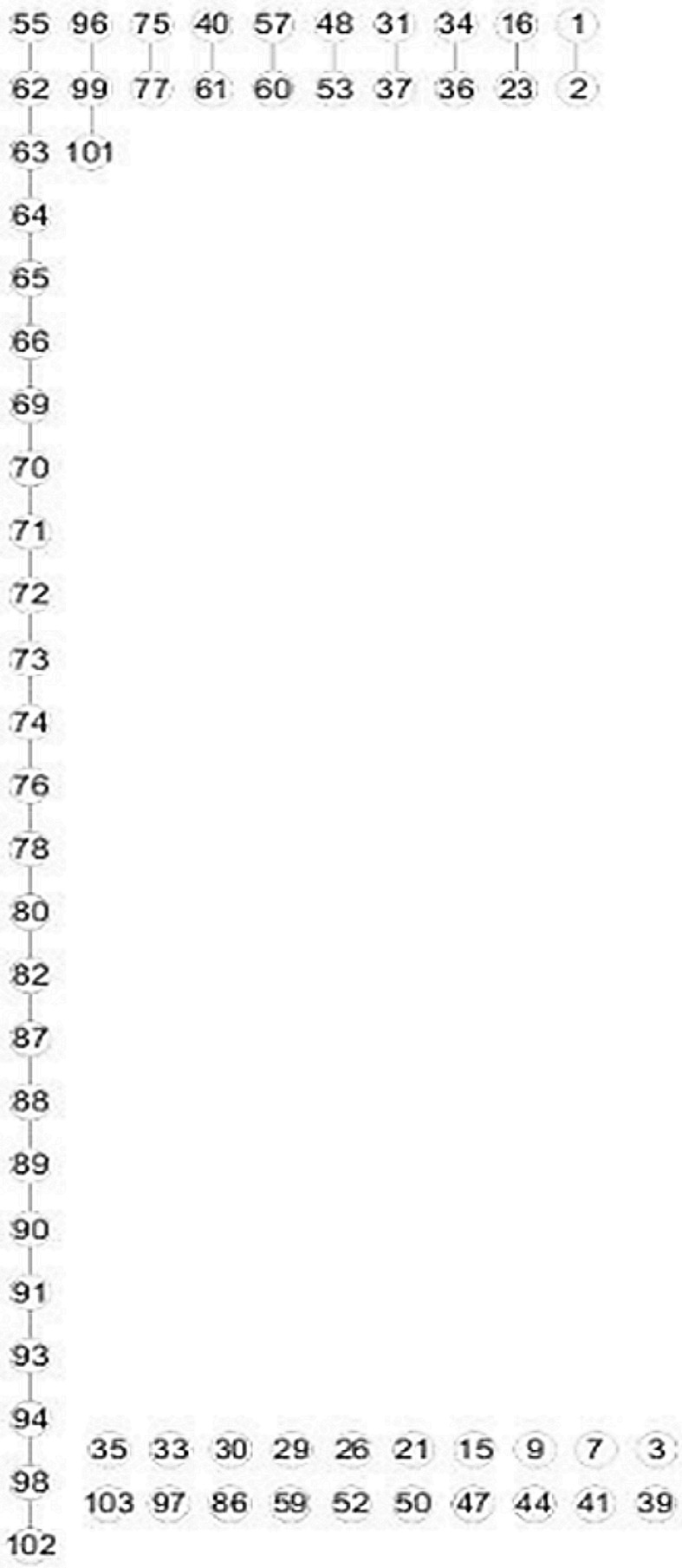
An adjusted PGM obtained by further applying frequentist diagonal ordering to the adjacency matrix of the PGM shown in Fig. 3b

Instead of being restricted to the 20 representative markers, we then investigated the set of 64 unique markers on Chr.5 after missing data imputation. A graphical display of all pairwise recombination frequencies implied that some of the 64 markers were genetically closely or completely coinciding (Fig. S5a). Results of five independent mapping runs in JoinMap 4.1 further showed that the majority of genetically similar markers were located on the first half of Chr.5 and they led to chaos in the ordering of markers (Fig. S5b). An MST and a PGM were constructed, respectively, from the same set of marker data (Fig. 3; a linearized version of the MST is shown in Fig.S6). The connectivity patterns revealed in the two graphs were generally similar to each other. Specifically, the lower parts of both graphs had roughly vertical linear structures, whereas the upper parts expanded horizontally instead of vertically and there was no obvious clue to the linearity of markers in this region. By further applying frequentist diagonal ordering to the adjacency matrix of the PGM, we obtained the graph shown in Fig. 4. The long string on the left of the graph clearly indicated the linearity of markers at the second half of Chr.5. The short strings at the upper right of the graph were mainly extracted from the nested part of the PGM, i.e. the first half of Chr.5. Though each of the short strings revealed, to some extent, the linearity between a couple of markers, as a whole they failed to form a coherent string and thus, were, not very informative to an accurate map construction. Isolated markers at the lower right of the graph should be excluded from map construction anyway, because of the fact that they occurred either with big genotyping errors or they were genetically very similar to other markers. Again, a series of linkage maps were constructed by sequentially deleting the markers with the highest, positive N.N.Stress (Fig. S7). Not surprisingly, the deleted markers shown at the top of Fig. S7 overlapped, to a large extent, with those markers excluded from the long string in Fig. 4. It is worth noting that in addition to the first half of Chr.5, a few other problematic markers on the second part of this chromosome, i.e. markers 75, 77, 86 and 97, were also unanimously diagnosed by all three approaches.

### Barley data

#### Linkage map construction involving a reciprocal translocation

**Fig. 5:**
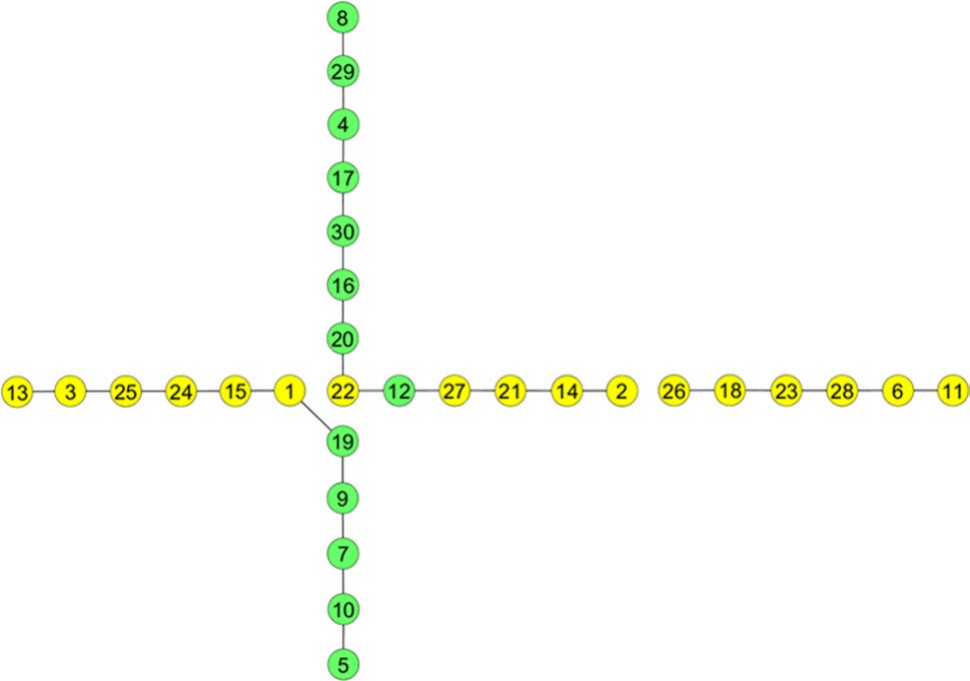
A PGM constructed from the barley data by the PC-stable algorithm in combination with frequentist diagonal ordering. *Yellow* nodes stand for markers on chromosome 1H and *green* nodes stand for markers on chromosome 3H. The significance level for conditional independence tests was set at 0.05

Figure 5 presents the PGM constructed from the barley data by the PC-stable algorithm in combination with frequentist diagonal ordering. Impressively, instead of finding a ‘pseudo-linkage’ between markers of chromosomes 1H and 3H, as obtained with standard methods, we obtained a cross-like configuration between the given markers. The translocation breakpoint was located around markers 12, 19, 20 that belong to chromosome 1H and markers 1, 22 that belong to chromosome 3H. Moreover, markers on the distal parts of the two chromosomes were perfectly linearly ordered. Our findings were in full agreement with the reference map (Table S2) supplied by the data providers.

## Discussion

Our proposed method in principle can be applied to linkage mapping involving large numbers of markers. More generally, whatever the number of markers is, a three-step framework for achieving an accurate genetic map is as follows. First, cut up the set of markers into a number of linkage groups corresponding to the number of a single set of chromosomes. Second, for markers within a single linkage group, whatever the size, use K-medoids clustering to produce a limited set of clusters corresponding to the number of markers required for a framework map for that linkage group. Probably best to define the number of clusters slightly larger than the number of markers required for the framework map. This makes it possible to throw out clusters that are small or consist of isolated markers. Third, take the cluster centres, i.e. representative markers, of the larger groups, and start with the construction of PGMs at that point.

We have shown through the barley example that it is possible to simultaneously realize marker grouping and ordering with PGMs, which are constructed through a series of well-structured conditional independence tests, e.g. the PC-stable algorithm. Of course, the estimated number of linkage groups is subject to the significance level *α* adopted in the conditional independence tests. Empirically, smaller values of *α* tend to lead to sparser graphs (Colombo and Maathuis 2014) that are equivalent to conservative grouping of markers, i.e. more linkage groups of smaller size.

By definition, the map distance is measured in cM; 1 cM approximately corresponds to 1% recombination frequency. Constructing PGMs from the observed genotype data involves the calculation of partial correlation coefficients, which essentially measure the combined effect of recombination frequencies between all markers, error rates of markers and marker order. Consequently, once marker grouping and ordering have been achieved using PGMs, one still has to calculate recombination frequencies and genetic distances to obtain a complete genetic map.

Like most existing approaches to linkage map construction, our method is based on the assumption of independent recombination events. In reality, however, chiasma interference (hereafter simply referred to as interference) occurs when the occurrence of one crossover (or chiasma) influences the probability of another crossover occurring nearby, especially in regions of high marker density (Weeks et al. 1994). Assuming no interference simplifies the construction of linkage maps but it leads to considerable overestimation of map distances (Speed and Waterman 1996). In contrast to Haldane’s mapping function that is applicable in the absence of interference, Kosambi’s mapping function has been invented and empirically verified to well describe the mathematical relation between recombination frequency and map distance in the case of interference. And yet, the performance of PGMs in constructing linkage maps in the face of interference together with data perturbations caused by genotyping errors and reciprocal translocations is currently unclear and deserves further investigation.

A few other studies have also applied graph-theoretic approaches to genetic map construction for plant species (Ronin et al. 2012; Wu et al. 2008a; Yap et al. 2003). However, they all concentrated on map integration, aiming at producing a consensus genetic map using maps from different populations. We have shown that PGMs present great potential for constructing a reliable genetic map for a single population, by constructing a genetic map in combination with tackling problems that are caused by genotyping errors and reciprocal translocations in the data.

### Author contribution statement

FE conceived the study. HW conducted the theoretical derivation. HW and JJ analysed the data and interpreted the results. HW, FE and JJ wrote the paper. All authors have read and approved the final manuscript.

## Electronic supplementary material

Below is the link to the electronic supplementary material.

Supplementary material 1 (DOCX 1743 kb)
