# Supplementary material for: The potential of probabilistic graphical models in linkage map construction
Source: Theor Appl Genet. 2016 Dec 5;130(2):433–44. doi: 10.1007/s00122-016-2824-x (PMC5263214; doi:10.1007/s00122-016-2824-x)

1. **Detailed mathematical derivation**

When *ε_M1_* = *ε_M3_* = 0 and *ε_M2_* = *ε*, *r_M1M2_* = (1–2*θ*_12_)(1–2*ε*), *r_M2M3_* = (1–2*θ*_23_)(1–2*ε*) and *r_M1M3_* = (1–2*θ*_12_)(1–2*θ*_23_).

Let *a* = 1–2*θ*_12_, *b* = 1–2*θ*_23_, *x* = 1–2*ε*.

∵ 0 < *θ*_12_ < 0.5, ∴ 0 < *a* < 1

∵ 0 < *θ*_23_ < 0.5, ∴ 0 < *b* < 1

∵ 0 < *ε* < 0.5, ∴ 0 < *x* < 1

$$\rho_{M1M2|M3}=\frac{r_{M1M2}-r_{M1M3}\times r_{M2M3}}{\sqrt{1-r_{M1M3}^{2}}\sqrt{1-r_{M2M3}^{2}}}$$

$$=\frac{\left(1-2\theta_{12}\right)\left(1-2\varepsilon\right)-\left(1-2\theta_{12}\right)\left(1-2\theta_{23}\right)\left(1-2\theta_{23}\right)\left(1-2\varepsilon\right)}{\sqrt{1-\left(1-2\theta_{12}\right)^{2}\left(1-2\theta_{23}\right)^{2}}\sqrt{1-\left(1-2\theta_{23}\right)^{2}\left(1-2\varepsilon\right)^{2}}}$$

$$=\frac{ax-ab^{2}x}{\sqrt{1-a^{2}b^{2}}\sqrt{1-b^{2}x^{2}}}=\frac{a\left(1-b^{2}\right)}{\sqrt{1-a^{2}b^{2}}}\left(x^{-2}-b^{2}\right)^{-\frac{1}{2}}$$

∵ $\frac{d\rho_{M1M2|M3}}{dx}=\frac{a\left(1-b^{2}\right)}{\sqrt{1-a^{2}b^{2}}}\left(-\frac{1}{2}\right)\left(x^{-2}-b^{2}\right)^{-\frac{3}{2}}\left(-2\right)x^{-3}=\frac{a\left(1-b^{2}\right)}{\sqrt{1-a^{2}b^{2}}}\left(1-b^{2}x^{2}\right)^{-\frac{3}{2}}>0$

∴$\rho_{M1M2|M3}$is a monotonically increasing function of *x*.

Considering that *x* is a monotonically decreasing function of *ε*, $\rho_{M1M2|M3}$ is therefore a monotonically decreasing function of *ε*.

When *ε_M1_* = *ε_M3_* = 0 and *ε_M2_* = *ε*, *r_M1M2_* = (1–2*θ*_12_)(1–2*ε*), *r_M2M3_* = (1–2*θ*_23_)(1–2*ε*) and *r_M1M3_* = (1–2*θ*_12_)(1–2*θ*_23_).

Let *a* = 1–2*θ*_12_, *b* = 1–2*θ*_23_, *x* = (1–2*ε*)^2^.

∵ 0 < *θ*_12_ < 0.5, ∴ 0 < *a* < 1

∵ 0 < *θ*_23_ < 0.5, ∴ 0 < *b* < 1

∵ 0 < *ε* < 0.5, ∴ 0 < *x* < 1

$$\rho_{M1M3|M2}=\frac{r_{M1M3}-r_{M1M2}\times r_{M2M3}}{\sqrt{1-r_{M1M2}^{2}}\sqrt{1-r_{M2M3}^{2}}}$$

$$=\frac{\left(1-2\theta_{12}\right)\left(1-2\theta_{23}\right)-\left(1-2\theta_{12}\right)\left(1-2\varepsilon\right)\left(1-2\theta_{23}\right)\left(1-2\varepsilon\right)}{\sqrt{1-\left(1-2\theta_{12}\right)^{2}\left(1-2\varepsilon\right)^{2}}\sqrt{1-\left(1-2\theta_{23}\right)^{2}\left(1-2\varepsilon\right)^{2}}}$$

$$=\frac{ab-abx}{\sqrt{1-a^{2}x}\sqrt{1-b^{2}x}}=ab\left(1-x\right)\left[\left(1-a^{2}x\right)\left(1-b^{2}x\right)\right]^{-\frac{1}{2}}$$

$$\frac{d\rho_{M1M3|M2}}{dx}=-ab\left[\left(1-a^{2}x\right)\left(1-b^{2}x\right)\right]^{-\frac{1}{2}}+ab\left(1-x\right)\left(-\frac{1}{2}\right)\left[\left(1-a^{2}x\right)\left(1-b^{2}x\right)\right]^{-\frac{3}{2}}\left[\left(-a^{2}\right)\left(1-b^{2}x\right)-b^{2}\left(1-a^{2}x\right)\right]$$

$$=-ab\left[\left(1-a^{2}x\right)\left(1-b^{2}x\right)\right]^{-\frac{1}{2}}+ab\left(1-x\right)\frac{1}{2}\left[\left(1-a^{2}x\right)\left(1-b^{2}x\right)\right]^{-\frac{3}{2}}\left[a^{2}\left(1-b^{2}x\right)+b^{2}\left(1-a^{2}x\right)\right]$$

$$=ab\left[\left(1-a^{2}x\right)\left(1-b^{2}x\right)\right]^{-\frac{1}{2}}\left\{-1+\frac{1}{2}\left(1-x\right)\left[\left(1-a^{2}x\right)\left(1-b^{2}x\right)\right]^{-1}\left[a^{2}\left(1-b^{2}x\right)+b^{2}\left(1-a^{2}x\right)\right]\right\}$$

$$=ab\left[\left(1-a^{2}x\right)\left(1-b^{2}x\right)\right]^{-\frac{1}{2}}\left\{-1+\frac{1}{2}\left(1-x\right)\frac{a^{2}\left(1-b^{2}x\right)+b^{2}\left(1-a^{2}x\right)}{\left(1-a^{2}x\right)\left(1-b^{2}x\right)}\right\}$$

$$=ab\left[\left(1-a^{2}x\right)\left(1-b^{2}x\right)\right]^{-\frac{1}{2}}\left\{-1+\frac{1}{2}\left(1-x\right)\left[\frac{a^{2}}{1-a^{2}x}+\frac{b^{2}}{1-b^{2}x}\right]\right\}$$

$$=ab\left[\left(1-a^{2}x\right)\left(1-b^{2}x\right)\right]^{-\frac{1}{2}}\left\{-1+\frac{1}{2}\left[\frac{a^{2}\left(1-x\right)}{1-a^{2}x}+\frac{b^{2}\left(1-x\right)}{1-b^{2}x}\right]\right\}$$

$$<ab\left[\left(1-a^{2}x\right)\left(1-b^{2}x\right)\right]^{-\frac{1}{2}}\left\{-1+\frac{1}{2}\left(a^{2}+b^{2}\right)\right\}$$

$$<ab\left[\left(1-a^{2}x\right)\left(1-b^{2}x\right)\right]^{-\frac{1}{2}}\left\{-1+\frac{1}{2}\times2\right\}=0$$

Thus, $\rho_{M1M2|M3}$is a monotonically decreasing function of *x*. Considering that *x* is a monotonically decreasing function of *ε*, $\rho_{M1M2|M3}$ is accordingly a monotonically increasing function of *ε*.

1. **Supplemental figures**

**Fig.S1** The proportion of heterozygous scores for individuals in the cucumber data set.

**Fig.S2** Five genetic maps generated repeatedly by the ML algorithm of JoinMap for a linkage group consisting of 340 markers that was obtained with a threshold of 0.15 for the recombination frequency. According to the consistent (small) gap at approx. 360 cM, this linkage group could be split into two linkage groups of 177 and 163 markers, respectively.

**Fig.S3** Linearized MST for 20 representative markers of Chr.5. The numbers between connected markers represent the number of recombinations and simple matching coefficient of similarity, respectively.


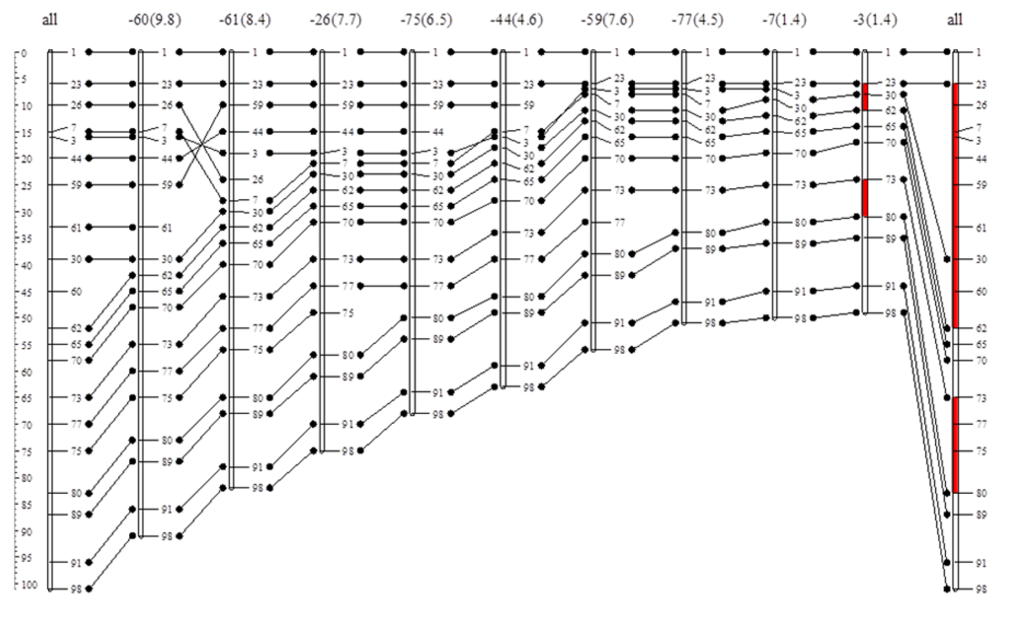


**Fig.S4** Linkage maps of Chr.5 obtained by sequentially deleting markers with the highest, positive N.N.Stress from the set of 20 representative markers. The deleted markers are shown above the linkage maps; the associated N.N.Stress is given between brackets. Comparison of the last two maps indicates regions (shown in red) where markers have been deleted and the associated reductions in map length.


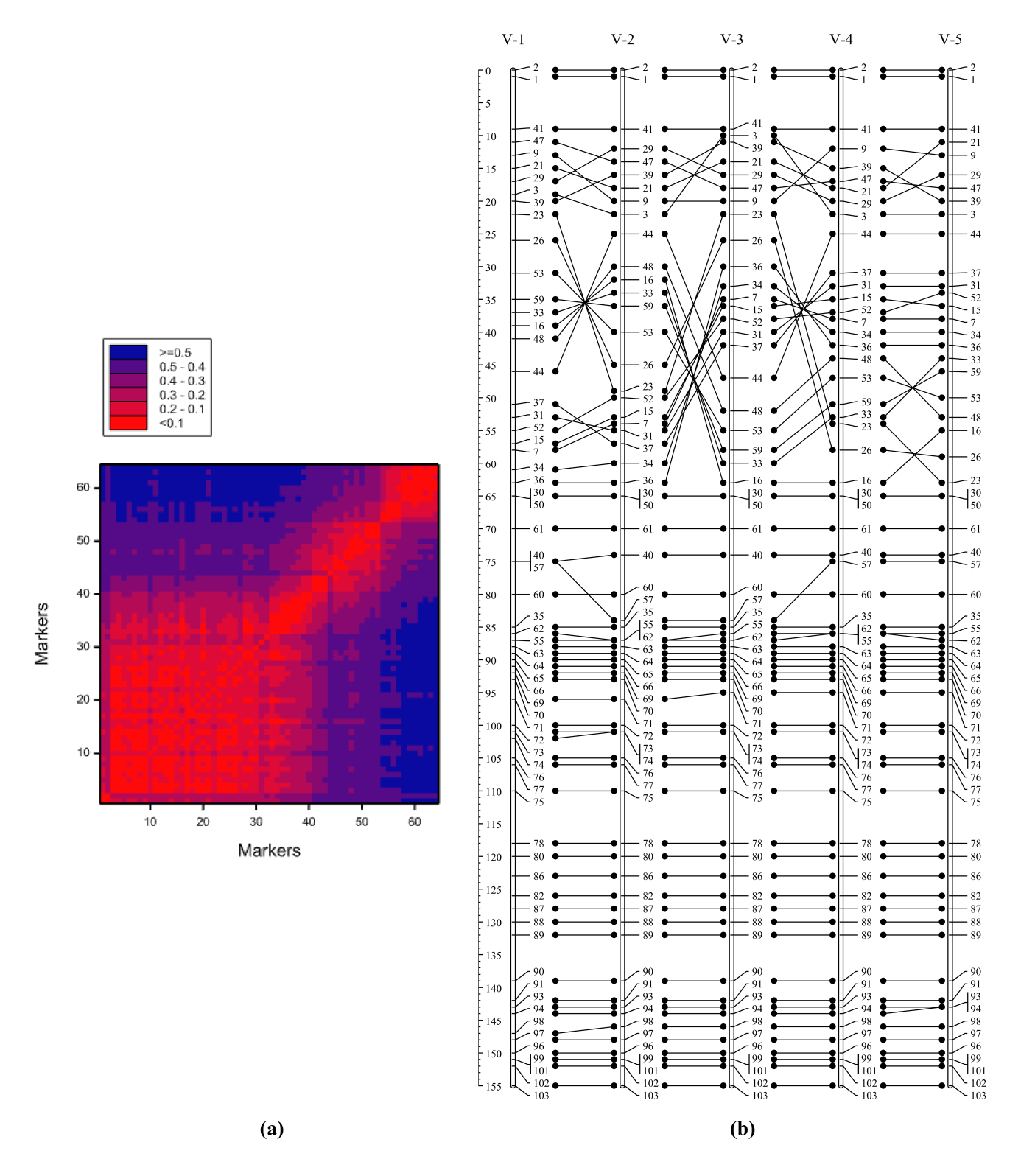


**Fig.S5 (a)** Pairwise recombination frequencies estimated for 64 unique markers of Chr.5. Markers are sorted according to their numerical labels. **(b)** Five linkage maps obtained by independent mapping runs in JoinMap 4.1 for the 64 markers.

**Fig.S6** Linearized MST for 64 unique markers of Chr.5.


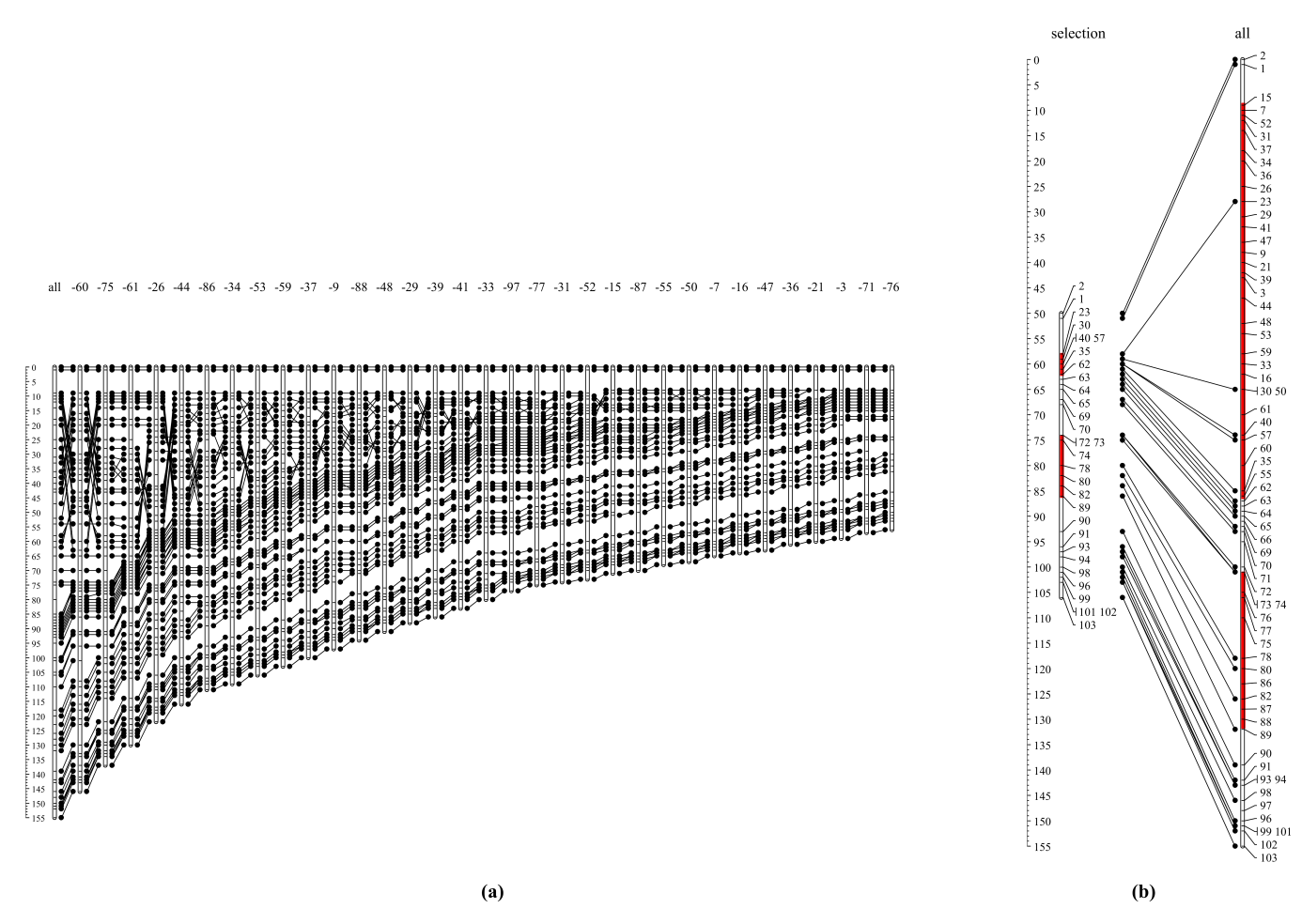


**Fig.S7** Linkage maps obtained by sequentially deleting the unique markers with the highest, positive N.N.Stress on Chr.5. The deleted markers are shown above the linkage maps.

1. **Supplemental tables**

**Table S1** The map position and the N.N.Stress of each marker obtained with JoinMap 4.1 from the simulated marker data.

| Nr | Locus | Position (cM) | N.N. Stress (cM) |
| --- | --- | --- | --- |
| 1 | marker1 | 0 |  |
| 2 | marker2 | 1.01 | -0.028 |
| 3 | marker3 | 2.362 | -0.047 |
| 4 | marker4 | 4.057 | -0.023 |
| 5 | marker5 | 4.728 | -0.014 |
| 6 | marker6 | 5.738 | -0.028 |
| 7 | marker7 | 7.09 | -0.057 |
| 8 | marker8 | 9.131 | -0.057 |
| 9 | marker9 | 10.482 | -0.009 |
| 10 | marker10 | 10.817 | -0.012 |
| 11 | marker11 | 12.512 | -0.059 |
| 12 | marker12 | 14.207 | -0.035 |
| 13 | marker13 | 15.217 | -0.014 |
| 14 | marker14 | 15.888 | -0.014 |
| 15 | marker15 | 16.898 | -0.028 |
| 16 | marker16 | 18.25 | -0.047 |
| 17 | marker17 | 19.945 | -0.023 |
| 18 | marker18 | 20.616 | 0.671 |
| 19 | marker19 | 21.287 | -0.028 |
| 20 | marker20 | 23.328 | -0.072 |
| 21 | marker21 | 25.023 | -0.047 |
| 22 | marker22 | 26.375 | -0.047 |
| 23 | marker23 | 28.07 | -0.023 |
| 24 | marker24 | 28.741 | -0.023 |
| 25 | marker25 | 30.436 | -0.023 |
| 26 | marker26 | 31.107 | -0.023 |
| 27 | marker27 | 32.802 | -0.023 |
| 28 | marker28 | 33.473 | -0.019 |
| 29 | marker29 | 34.825 | -0.077 |
| 30 | marker30 | 37.565 | -0.019 |
| 31 | marker31 | 37.9 | -0.007 |
| 32 | marker32 | 38.91 | -0.05 |
| 33 | marker33 | 41.299 | -0.102 |
| 34 | marker34 | 43.34 | 2.041 |
| 35 | marker35 | 45.73 | -0.102 |
| 36 | marker36 | 47.771 | -0.014 |
| 37 | marker37 | 48.105 | -0.012 |
| 38 | marker38 | 49.8 | -0.047 |
| 39 | marker39 | 51.152 | -0.047 |
| 40 | marker40 | 52.847 | -0.023 |
| 41 | marker41 | 53.518 | -0.023 |
| 42 | marker42 | 55.213 | -0.035 |
| 43 | marker43 | 56.223 | -0.035 |
| 44 | marker44 | 57.918 | -0.023 |
| 45 | marker45 | 58.59 | -0.019 |
| 46 | marker46 | 59.941 | -0.028 |
| 47 | marker47 | 60.951 | -0.028 |
| 48 | marker48 | 62.303 | -0.038 |
| 49 | marker49 | 63.654 | -0.038 |
| 50 | marker50 | 65.005 | -0.152 |
| 51 | marker51 | 70.273 | 5.629 |
| 52 | marker52 | 74.443 | -0.059 |
| 53 | marker53 | 75.114 | -0.009 |
| 54 | marker54 | 75.785 | -0.033 |
| 55 | marker55 | 78.174 | -0.033 |
| 56 | marker56 | 78.846 | 0.671 |
| 57 | marker57 | 80.887 | -0.057 |
| 58 | marker58 | 82.238 | -0.047 |
| 59 | marker59 | 83.933 | -0.047 |
| 60 | marker60 | 85.285 | -0.019 |
| 61 | marker61 | 85.956 | -0.009 |
| 62 | marker62 | 86.627 | -0.142 |
| 63 | marker63 | 96.145 | 11.01 |
| 64 | marker64 | 102.536 | -0.139 |
| 65 | marker65 | 103.546 | 0.666 |
| 66 | marker66 | 104.898 | -0.009 |
| 67 | marker67 | 105.232 | -0.005 |
| 68 | marker68 | 105.903 | -0.014 |
| 69 | marker69 | 106.914 | -0.028 |
| 70 | marker70 | 108.265 | -0.047 |
| 71 | marker71 | 109.96 | -0.047 |
| 72 | marker72 | 111.312 | -0.077 |
| 73 | marker73 | 114.052 | -0.118 |
| 74 | marker74 | 116.093 | -0.043 |
| 75 | marker75 | 117.103 | -0.035 |
| 76 | marker76 | 118.798 | -0.072 |
| 77 | marker77 | 120.839 | -0.087 |
| 78 | marker78 | 122.88 | 0.642 |
| 79 | marker79 | 124.576 | -0.023 |
| 80 | marker80 | 125.247 | -0.019 |
| 82 | marker82 | 126.598 | -0.009 |
| 81 | marker81 | 126.933 | 0.688 |
| 83 | marker83 | 130.026 | -0.043 |
| 84 | marker84 | 130.697 | -0.019 |
| 85 | marker85 | 132.049 | -0.047 |
| 86 | marker86 | 133.744 | -0.023 |
| 87 | marker87 | 134.415 | -0.023 |
| 88 | marker88 | 136.11 | -0.035 |
| 89 | marker89 | 137.12 | -0.043 |
| 90 | marker90 | 139.161 | -0.043 |
| 91 | marker91 | 140.172 | -0.043 |
| 92 | marker92 | 142.213 | -0.043 |
| 93 | marker93 | 143.223 | -0.021 |
| 94 | marker94 | 144.233 | -0.043 |
| 95 | marker95 | 146.274 | -0.028 |
| 96 | marker96 | 146.945 | -0.019 |
| 97 | marker97 | 148.297 | -0.057 |
| 98 | marker98 | 150.338 | -0.072 |
| 99 | marker99 | 152.033 | -0.035 |
| 100 | marker100 | 153.043 | -0.05 |
| 101 | marker101 | 155.432 | 0.597 |
| 102 | marker102 | 158.173 | -0.159 |
| 103 | marker103 | 160.913 | -0.159 |
| 104 | marker104 | 163.654 | -0.058 |
| 105 | marker105 | 164.664 | -0.021 |
| 106 | marker106 | 165.674 | -0.014 |
| 107 | marker107 | 166.345 | -0.043 |
| 108 | marker108 | 169.439 | -0.228 |
| 109 | marker109 | 172.889 | -0.048 |
| 110 | marker110 | 173.56 | -0.009 |
| 111 | marker111 | 174.231 | -0.023 |
| 112 | marker112 | 175.926 | -0.012 |
| 113 | marker113 | 176.26 | -0.007 |
| 114 | marker114 | 177.271 | -0.028 |
| 115 | marker115 | 178.622 | -0.019 |
| 116 | marker116 | 179.293 | -0.014 |
| 117 | marker117 | 180.303 | -0.014 |
| 118 | marker118 | 180.974 | -0.023 |
| 119 | marker119 | 182.669 | -0.023 |
| 120 | marker120 | 183.341 | -0.009 |
| 121 | marker121 | 184.012 | -0.033 |
| 122 | marker122 | 186.401 | -0.05 |
| 123 | marker123 | 187.411 | 0.671 |
| 124 | marker124 | 188.083 | -0.038 |
| 125 | marker125 | 190.823 | -0.058 |
| 126 | marker126 | 191.833 | -0.035 |
| 127 | marker127 | 193.528 | -0.059 |
| 128 | marker128 | 195.223 | 2.046 |
| 129 | marker129 | 197.964 | -0.118 |
| 130 | marker130 | 200.005 | -0.043 |
| 131 | marker131 | 201.015 | -0.014 |
| 132 | marker132 | 201.686 | -0.028 |
| 133 | marker133 | 203.727 | -0.043 |
| 134 | marker134 | 204.737 | -0.021 |
| 135 | marker135 | 205.747 | 0.662 |
| 136 | marker136 | 207.789 | -0.043 |
| 137 | marker137 | 208.799 | -0.05 |
| 138 | marker138 | 211.188 | -0.067 |
| 139 | marker139 | 212.54 | -0.028 |
| 140 | marker140 | 213.55 | -0.014 |
| 141 | marker141 | 214.221 | -0.038 |
| 142 | marker142 | 216.961 | -0.019 |
| 143 | marker143 | 217.296 | -0.005 |
| 144 | marker144 | 217.967 | -0.023 |
| 145 | marker145 | 219.662 | -0.137 |
| 146 | marker146 | 223.47 | -0.109 |
| 147 | marker147 | 224.822 | -0.038 |
| 148 | marker148 | 226.173 | -0.038 |
| 149 | marker149 | 227.524 | 0.647 |
| 150 | marker150 | 229.914 | 0.584 |
| 151 | marker151 | 233.008 | -0.11 |
| 152 | marker152 | 234.703 | -0.097 |
| 153 | marker153 | 237.443 | -0.058 |
| 154 | marker154 | 238.453 | -0.105 |
| 155 | marker155 | 243.352 | 6.318 |
| 156 | marker156 | 247.16 | 0.51 |
| 157 | marker157 | 250.254 | -0.065 |
| 158 | marker158 | 251.264 | -0.021 |
| 159 | marker159 | 252.274 | -0.021 |
| 160 | marker160 | 253.285 | -0.089 |
| 161 | marker161 | 257.454 | -0.119 |
| 162 | marker162 | 258.805 | -0.028 |
| 163 | marker163 | 259.815 | -0.028 |
| 164 | marker164 | 261.167 | -0.057 |
| 165 | marker165 | 263.208 | -0.087 |
| 166 | marker166 | 265.249 | -0.072 |
| 167 | marker167 | 266.944 | -0.047 |
| 168 | marker168 | 268.295 | -0.047 |
| 169 | marker169 | 269.99 | -0.084 |
| 170 | marker170 | 272.38 | -0.067 |
| 171 | marker171 | 273.731 | -0.038 |
| 172 | marker172 | 275.083 | -0.009 |
| 173 | marker173 | 275.417 | -0.009 |
| 174 | marker174 | 276.769 | 0.666 |
| 175 | marker175 | 277.779 | -0.043 |
| 176 | marker176 | 279.82 | -0.057 |
| 177 | marker177 | 281.171 | -0.019 |
| 178 | marker178 | 281.843 | -0.019 |
| 179 | marker179 | 283.194 | -0.038 |
| 180 | marker180 | 284.545 | -0.057 |
| 181 | marker181 | 286.587 | 0.642 |
| 182 | marker182 | 288.282 | -0.023 |
| 183 | marker183 | 288.953 | -0.116 |
| 184 | marker184 | 296.883 | 10.939 |
| 185 | marker185 | 304.424 | -0.281 |
| 186 | marker186 | 306.119 | -0.084 |
| 187 | marker187 | 308.509 | -0.102 |
| 188 | marker188 | 310.55 | -0.028 |
| 189 | marker189 | 311.221 | -0.048 |
| 190 | marker190 | 314.671 | 0.612 |
| 191 | marker191 | 316.366 | -0.097 |
| 192 | marker192 | 319.106 | -0.159 |
| 193 | marker193 | 321.847 | -0.138 |
| 194 | marker194 | 324.236 | -0.213 |
| 195 | marker195 | 328.405 | 0.621 |
| 196 | marker196 | 329.757 | -0.038 |
| 197 | marker197 | 331.108 | -0.009 |
| 198 | marker198 | 331.442 | -0.019 |
| 199 | marker199 | 334.183 | 0.612 |
| 200 | marker200 | 336.224 |  |

**Table S2** The neighbourhood obtained by the lasso approach for the 30 markers on chromosomes 1H and 3H of the barley data. An ‘x’ symbol indicates that the corresponding row marker is strongly linked to the column marker. Markers are ordered so that both chromosomes are linearly structured, and meanwhile the translocation breakpoint is clearly displayed off-diagonal.


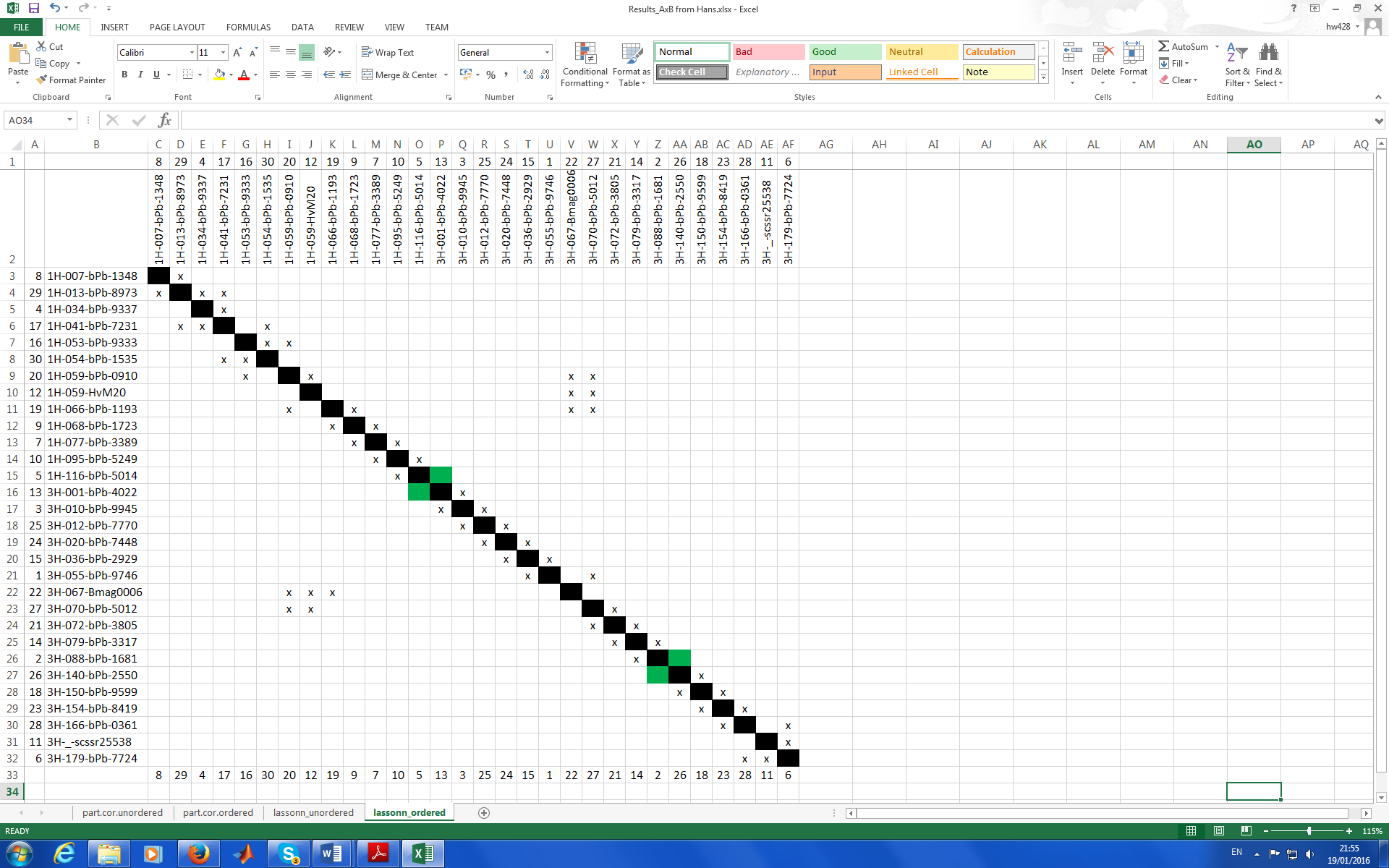

Supplement: Supplementary file 1 — Supplementary material 1 (DOCX 1743 kb) [file 122_2016_2824_MOESM1_ESM.docx]
